# Behavioral, antioxidant, and kynurenine pathway modulation of a specific strain of *Ligilactobacillus salivarius* in a preclinical model of depression

**DOI:** 10.1007/s00394-026-03941-9

**Published:** 2026-03-09

**Authors:** David Martín-Hernández, Javier R. Caso, César Díaz-García, Pedro-Antonio Regidor, José Miguel Rizo, Marta Román, Rocío Gutiérrez, Juan Carlos Leza

**Affiliations:** 1 Department of Pharmacology and Toxicology, School of Medicine, Faculty of Medicine, Complutense University of Madrid (UCM), Hospital 12 de Octubre Research Institute (Imas12), Neurochemistry Research Institute UCM (IUIN), Spanish network in stress research (REIS), Madrid. Pza. Ramón y Cajal s/n, 28040 Madrid, Spain; 2 Biomedical Network Research Center of Mental Health, Institute of Health Carlos III (CIBERSAM, ISCIII), Madrid, Spain; 3 Exeltis Healthcare, Adalperostr. 84, 85737 Ismaning, Germany; 4 OTC Chemo, Manuel Pombo Angulo 28-4th Floor, 28050 Madrid, Spain

**Keywords:** *Ligilactobacillus salivarius*, depression, chronic mild stress, antidepressant, antioxidant, kynurenine metabolites

## Abstract

**Purpose:**

Current antidepressants targeting neurotransmitters often fail to alleviate symptoms. Alternative hypotheses suggest inflammation may trigger an alternative route that converts tryptophan into kynurenine, reducing the bioavailability of tryptophan to synthesize serotonin while producing neuroactive metabolites such as quinolinic acid (QUINA, excitotoxic) and kynurenic acid (KYNA, neuroprotective). This study evaluates the effects on these systems of a specific strain of *Ligilactobacillus salivarius* (*L. salivarius*), identified in the Spanish Type Culture Collection as CECT 30632, in a preclinical model depression.

**Methods:**

Male Wistar rats (*n* = 32) were divided into control (CT) and chronic mild stress (CMS) groups, treated with either vehicle or *L. salivarius* CECT 30632 for four weeks, starting one week before CMS exposure. Behavioral assessments, including the splash test (ST) and open field test (OF), were conducted. Biochemical analyses of peripheral blood mononuclear cells (PBMCs), plasma, and frontal cortex (FC) samples assessed antioxidant markers phospho-nuclear factor (erythroid-derived 2)-like 2 (p-Nrf2) and glutathione peroxidase 1 (GPx1), as well as tryptophan metabolites.

**Results:**

In the ST, *L. salivarius* CECT 30,632 reduced latency to groom, indicating improved anhedonia and self-care, while no changes were observed in the OF test. CMS reduced p-Nrf2 and GPx1 expression in PBMCs, which was restored by *L. salivarius* CECT 30,632. This bacterium also reduced the QUINA/KYNA ratio in plasma and FC, suggesting a lower excitotoxicity risk.

**Conclusion:**

*Ligilactobacillus salivarius* CECT 30632 improved behavioral outcomes, enhanced antioxidant defenses, and modulated tryptophan metabolism in a rat model of CMS. These findings support its potential as a probiotic intervention for depression.

**Supplementary Information:**

The online version contains supplementary material available at 10.1007/s00394-026-03941-9.

## Background

Nearly 333 million people worldwide (4.36%) suffer from depressive disorders, with this prevalence rising sharply to 5.94% in individuals over 70 years of age [1]. Depressive disorders encompass a heterogeneous spectrum of serious mental conditions that affect mood, cognition, and physiological functions [2]. In 2021, depression was ranked as the 13th leading contributor to the global burden of disease, accounting for 56 million Disability-Adjusted Life cycle Years (DALYs), a 63% increase since the year 2000. Moreover, substantial social and economic consequences are inevitably bound to the impact on public health of mental diseases [3].

Existing treatments for depression primarily target neurotransmitter imbalances, yet alternative hypotheses have emerged over recent decades to unravel the complex and multifactorial nature of depression [4]. Both clinical and preclinical studies have identified alterations in the immune system [5–7], oxidative stress [8], antioxidant enzymes [9], and kynurenine pathways [10]. Notably, antidepressant activity has been associated with the modulation of these systems [11, 12].

Oxidative stress and inflammation are two sides of the same coin serving as homeostatic mechanisms whose imbalance is detrimental and has been implicated in psychiatric diseases [13, 14]. The nuclear factor (erythroid-derived 2)-like 2 (Nrf2) is the master orchestrator of the antioxidant response. Upon activation, Nrf2 translocates to the nucleus where it binds to antioxidant response elements (ARE) sequences in the genome, promoting the transcription of phase II antioxidant enzymes such as glutathione peroxidase 1 (GPx1) [15].

Serotonin (5-HT), the primary neurotransmitter associated with antidepressant treatment, is derived from the essential amino acid tryptophan. However, inflammation may trigger an alternative route that converts tryptophan into kynurenine, reducing the bioavailability of tryptophan to synthesize 5-HT while producing some neuroactive metabolites [16]. Among them, quinolinic acid (QUINA) activates N-methyl-D-aspartate (NMDA) receptors, potentially leading to glutamate excitotoxicity, whereas kynurenic acid (KYNA) inhibits NMDA signaling and is considered neuroprotective.

The gut microbiome has demonstrated a wide array of ways to impact on the central nervous system (CNS), affecting behavior, collectively termed the microbiota-gut-brain (MGB) axis [17]. Disruptions in the gut microbiome have been documented in CNS disorders [18], particularly in depressive patients [19], often in conjunction with innate immune alterations [20]. Thus, prebiotic, probiotic, and synbiotic interventions are under evaluation as potential treatments for mental disorders, with meta-analyses demonstrating their effects on the MGB axis [21]. Among these, probiotics have yielded the most promising results in clinical studies [22]. Although evidence regarding the modulation of kynurenine pathways by probiotics is limited [23], some human studies suggest that certain species of *Lactobacillus* and *Bifidobacterium* exert antidepressant effects while decreasing kynurenine levels [24, 25] or altering 5-HT turnover [26]. 

*Ligilactobacillus salivarius* (*L. salivarius*) is a Gram-positive lactic acid bacterial strain naturally present in the healthy human gut microbiome, recognized for its potential health benefits [27]. *L. salivarius* has demonstrated clinical improvement, including accelerating recovery in a mouse model of colitis [28] and mitigating alcohol-induced damage [29]. The molecular mechanisms modulated by *L. salivarius* encompass the production of the anti-inflammatory interleukin-10 [28], upregulation of the antioxidant master regulator Nrf2 [29], antimicrobial activity [30], and regulatory effects on the host microbiome [31]. All this evidence underscores the effects involved in an eventual probiotic activity relevant to CNS disorders.

Given the malfunction of the MGB axis observed in preclinical models of stress-related psychiatric disorders such as anxiety and depression, we aimed to evaluate whether administration of a specific strain of *L. salivarius*, identified in the Spanish Type Culture Collection as CECT 30632, has antidepressant properties using the well-established chronic mild stress (CMS) model of depression in rats [32].

## Materials & methods

### Animals

This study adhered to the modified ARRIVE guidelines 2.0 for preclinical in vivo research [33] and to Spanish and European Union regulations (RD 53/2013 and EU Directive 2010/63/EU for animal experiments). The experimental protocol was approved by the proper administrative authorities (PROEX 087/18) and conducted at the UCM Animal Facility of the Complutense University of Madrid.

Male Wistar Hannover rats (HsdRccHan: Wist, Envigo, Spain), weighing approximately 300 g, were housed in a controlled environment with a constant temperature of 24 ± 2 °C and relative humidity of 70 ± 5%, under a 12-hour light‒dark cycle (lights on at 8:00 AM). The rats were acclimated to these conditions and handled daily for seven days before the experiments, with unrestricted ad libitum access to fresh filtered tap water and standard pellet chow (A04 SAFE, Scientific Animal Food and Engineering, Augy, France) throughout the experimental procedures, except as specified by the chronic mild stress (CMS) protocol.

### Experimental protocol

**Fig. 1:**
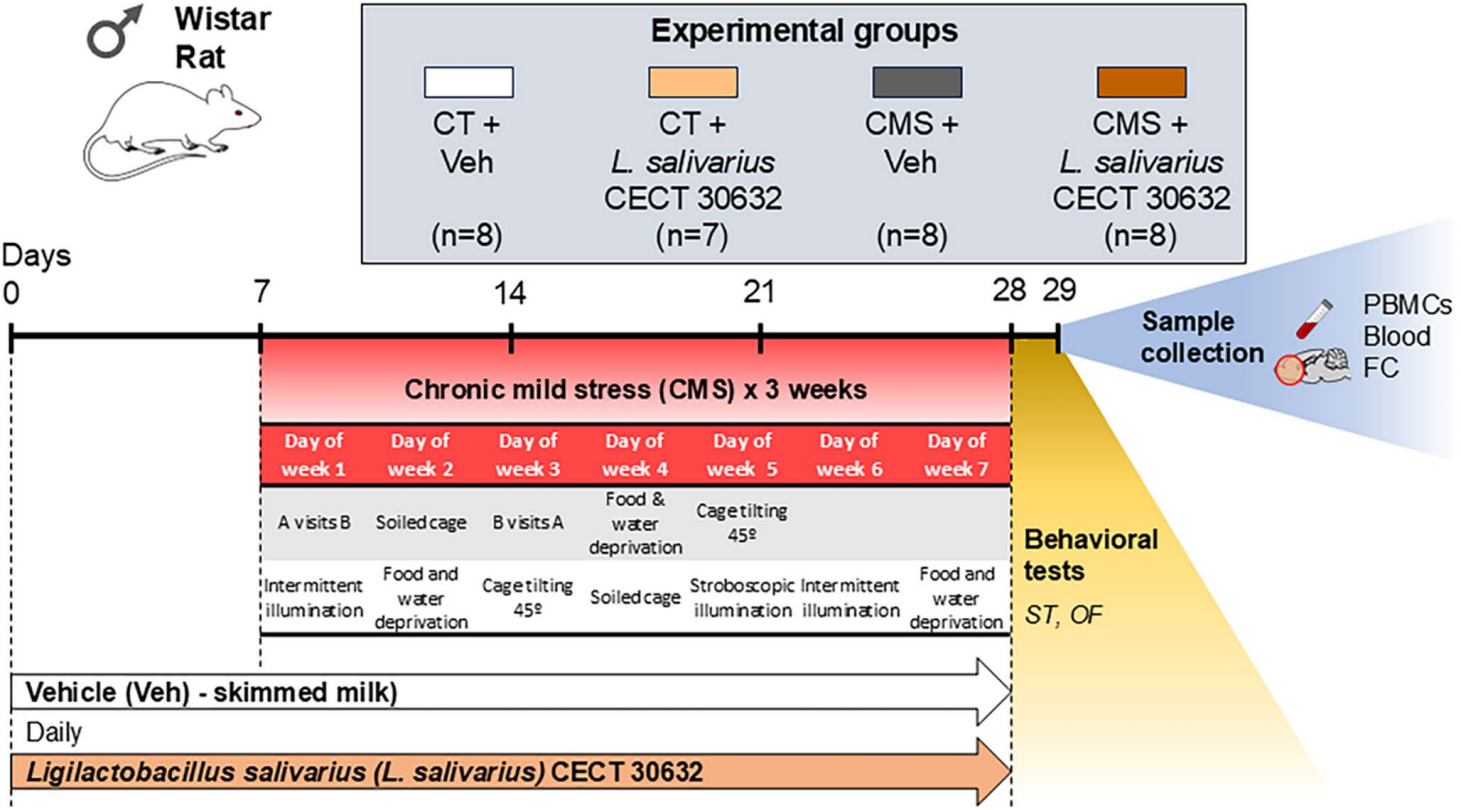
Experimental protocol. Schematic overview of the timeline, procedures, and groups generated by the experimental protocol. The figure was prepared using the Motifolio Illustration Toolkits (Motifolio Inc., Ellicott City, MD, USA)

Four experimental groups were formed by combining treatments with vehicle (Veh - placebo) or *L. salivarius* CECT 30632 and exposure to CMS or no exposure (control), totaling 32 male rats: CT + Veh (*n* = 8), CT + *L. salivarius* CECT 30632 (*n* = 7, one rat died during gavage), CMS + Veh (*n* = 8), and CMS + *L. salivarius* CECT 30632 (*n* = 8). Animals were randomly assigned to each group. Vehicle/placebo (skimmed milk, 1mL) or *L. salivarius* CECT 30632 (1 × 10^10^ colony forming units (CFU) dissolved in 1mL of skimmed milk) was administered daily by gavage for 4 weeks (days 1 to 28). The exposure to CMS began after 7 days of administration of *L. salivarius* CECT 30632 or the vehicle and continued until day 28 (3 weeks). Behavioral tests and sample collection were performed on day 29. Samples were collected between 2:00 and 3:00 PM following terminal anesthesia with sodium pentobarbital (220 mg/kg i.p. Vetoquinol^®^, Madrid, Spain) to minimize interference from circadian rhythms. (Fig. 1).

CMS encompasses a variety of stressors rotated unpredictably every 12 h, in an unavoidable, and uncontrollable manner. These stressors include [a] food deprivation, [b] water deprivation, [c] cage tilting, [d] soiled cages, [e] grouped housing after a period of water deprivation, [f] stroboscopic illumination [150 flashes/min], and [g] intermittent illumination every 2 h [34].

### Behavioral tests

Investigators were blinded when scoring behavioral tests. Moreover, videos were independently analyzed by two blinded researchers to minimize potential bias in behavioral evaluation.

#### Splash test (ST)

The ST measures parameters related to depressive-like behavior and was performed by adapting a protocol previously used [35]. The evaluation is carried out after day 21 of CMS through the grooming behavior of the animal after spraying a solution of 10% sucrose in the dorsal coat during the activity phase and under dark conditions. Grooming is considered a personal hygiene index, as well as an indirect measure of appetite for a sweet solution related to anhedonia. The time until the start of grooming (latency) and the total grooming time were quantified with the help of a camcorder for a period of 5 min and analyzed by researchers blinded to the experiment conditions. Latency time correlates positively with anhedonia and inversely with the self-care index, whereas total grooming time correlates inversely with anhedonia and positively with the self-care index.

#### Open field (OF)

Anxious behavior and locomotive activity were measured during the light phase (between 8 am and 8 pm) in a black square cabin (90 × 90 × 45 cm) in low light conditions, where the rats were placed individually in the center of the box for 5 min free examination. The route was cleaned with 70% ethanol between sessions to eliminate any influx of possible olfactory signals that could reach the animal. The sessions were videotaped and the video software ANYMAZE (Stoelting Europe, Dublin, Ireland) was used for the analysis. After defining the center as an interior square with 54-cm sides (18 cm from the walls) and the periphery as the remaining area of the cubicle, the following variables were measured: time spent in the periphery, total distance covered and average speed. More time spent in the periphery corresponds to higher anxious behavior. Total distance covered and average speed are locomotion parameters.

### Tissue specimens

Blood was obtained via cardiac puncture, anticoagulated with 1% w/v ethylenediaminetetraacetic acid (EDTA) (1 volume EDTA per 50 volumes blood), and centrifuged at 1500 rpm for 15 min to obtain plasma. Peripheral blood mononuclear cells (PBMCs) were separated using a Ficoll protocol. Brain was harvested following decapitation, and the frontal cortex (FC) was dissected. All samples (plasma, PBMCs, and the FC) were immediately frozen at − 80 °C and stored until use.

Total protein extracts were prepared from PBMCs and FC tissue after homogenization in 1× PBS (pH = 7) supplemented with a protease inhibitor cocktail (cOmplete Roche, Basel, Switzerland) and a phosphatase inhibitor cocktail (phosSTOP Roche, Basel, Switzerland), using a Tissue-Lyser LT (QIAGEN, Hilden, Germany) at 50/s for 4 min, followed by centrifugation at 12,000 rpm for 10 min. The supernatants served as the total homogenates and were immediately frozen at -80 °C until analysis.

### Western blot

Protein levels in the total homogenates were quantified using the Bradford method, which is based on the principle of protein-dye binding. 15 µg of protein were mixed with Laemmli sample buffer (Bio-Rad, Hercules, CA, USA), loaded and size-separated by 8% sodium dodecyl sulfate‒polyacrylamide gel electrophoresis (90 V), and transferred to nitrocellulose membranes using the Trans-Blot Turbo Transfer System (Bio-Rad, Hercules, CA, USA). The membranes were blocked for 1 h with Tris-buffered saline (TBS) containing 0.1% Tween 20 and 5% bovine serum albumin and subsequently incubated overnight at 4 °C with specific primary antibodies against p-Nrf2 and GPx-1 (Table S1). After washing, the membranes were incubated with anti-rabbit IgG-HRP secondary antibody (Table S1) at room temperature for 90 min. The membranes were developed using the ECL Prime^®^ kit (Cytiva Marlborough, MA, USA) according to the manufacturer’s instructions. Blots were imaged using a ChemiDoc™ (Bio-Rad^®^, Hercules, CA, USA) and quantified by densitometry with the Fiji ImageJ^®^ package. Densitometry data were obtained in arbitrary optical density units and expressed as a percentage of the CT + Veh group (100%). Multiple exposure times ensured linearity of band intensities. Beta-actin (A5441 Sigma, 1:10000) was used as the loading control.

### Enzyme-linked immunosorbent assay (ELISA)

Commercial ELISA kits were used following the manufacturer’s instructions to analyze tryptophan, kynurenine, and 5-HT (LDN, Nordhorn, Germany); QUINA and KYNA (Cloud-Clone Corp., Houston, TX, USA). These assays have been validated and successfully used in previous scientific publications of our group using rodent samples [36, 37]. Assay performance was verified by ensuring that sample dilutions fell within the linear range of detection, using dilution factors previously optimized and re-evaluated in the present study. All samples were analyzed in duplicate to ensure consistency and reliability. Absorbance was measured using the Synergy 2 microplate reader (BioTek^®^, USA), and data processing was performed with the built-in Gen5 Data Analysis Software (BioTek^®^, USA).

### Statistical analysis

Data are expressed as the mean ± standard error of the mean (SEM). Significant outliers were identified using the robust regression and outlier removal (ROUT) method. The normality of distribution was assessed with the Shapiro–Wilk test, and homogeneity of variance was evaluated using the Brown–Forsythe test. When the data showed a Gaussian distribution and equal variances, one-way ANOVA followed by Tukey’s *post hoc* test for multiple comparisons was applied. For data with unequal variances, the Brown–Forsythe ANOVA test followed by Dunnett’s T3 post hoc test was used. For non-normally distributed data, a nonparametric Kruskal–Wallis test with Dunn’s multiple comparisons was employed. A *p* value ≤ 0.05 was considered statistically significant. Data analysis was performed using GraphPad Prism 9 (GraphPad Software, San Diego, CA, USA).

## Results

### Behavioral effects of *L. salivarius* CECT 30632 in anhedonia, anxiety, and locomotion after CMS

**Fig. 2:**
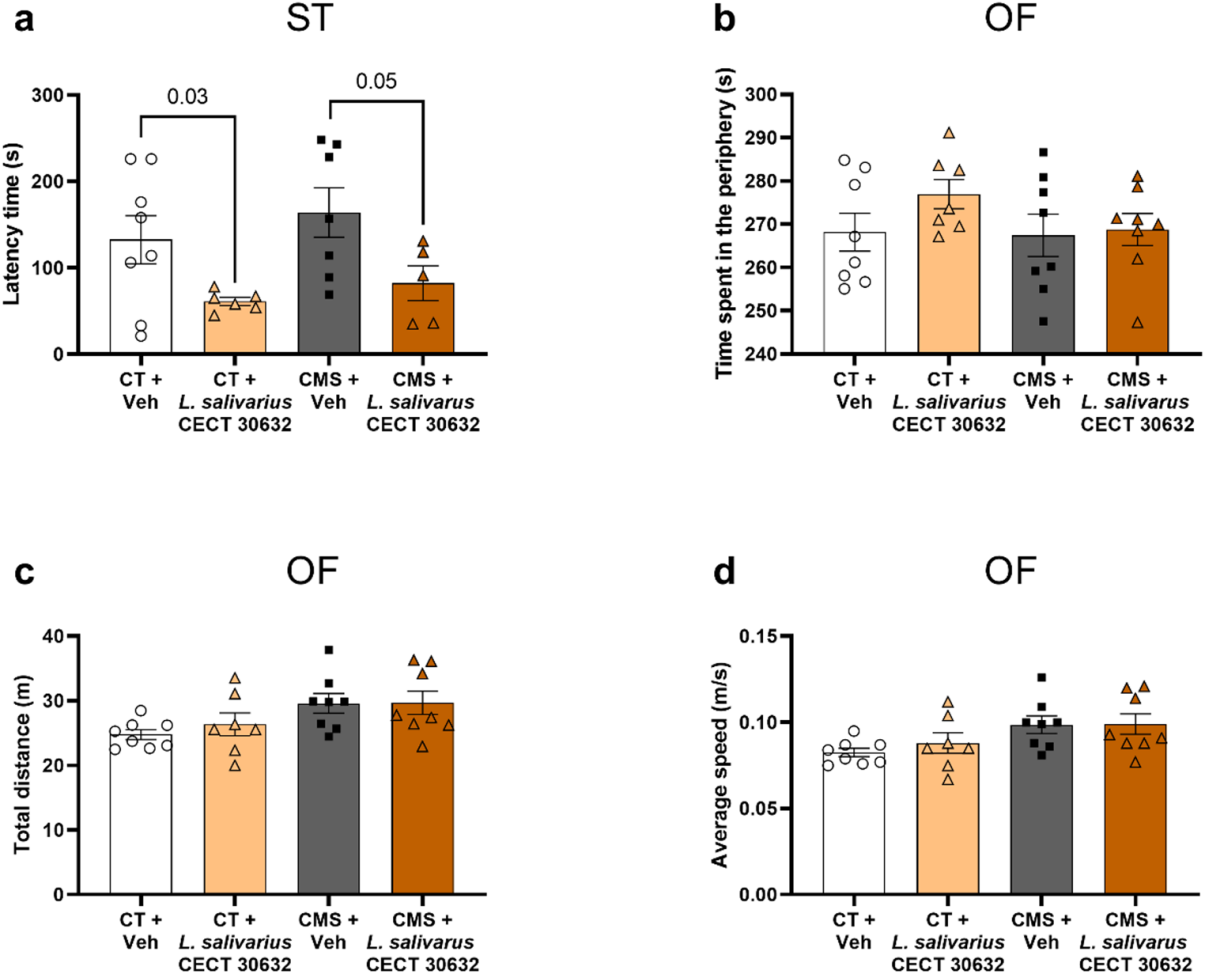
Behavioral effects of *L. salivarius* CECT 30632 after chronic mild stress (CMS) measured by the splash test (ST) and open field (OF). *L. salivarius* CECT 30632 decreases ST latency time (**a**), while no effects were observed on OF time spent in open arms (**b**), OF total distance (**c**), and OF average speed (**d**). The data are presented as the means ± SEMs. *p*-value of t-test paired comparisons are indicated with numbers. Brown–Forsythe ANOVA test followed by Dunnett’s T3 post hoc test (**a**), One-way ANOVA test followed by Tukey’s post hoc (**b-d**)

ST showed differences in latency time (*p* = 0.0235, Brown–Forsythe ANOVA test), with a clear trend to decrease in *L. salivarius* CECT 30632-treated groups (CT + Veh vs. CT + *L .salivarius* CECT 30632 *p* = 0.03, unpaired t test with Welch’s correction; CMS + Veh vs. CMS + *L. salivarius* CECT 30632 *p* = 0.05, unpaired t test) (Fig. 2a). Grooming time was similar among all experimental groups (data not shown, *p* = 0.1684, Brown–Forsythe ANOVA test). There were no alterations in OF for time spent in the periphery, total distance, and average speed (Fig. 2b-d).

### Antioxidant effects of *L. salivarius* CECT 30632 on PBMCs after CMS

**Fig. 3:**
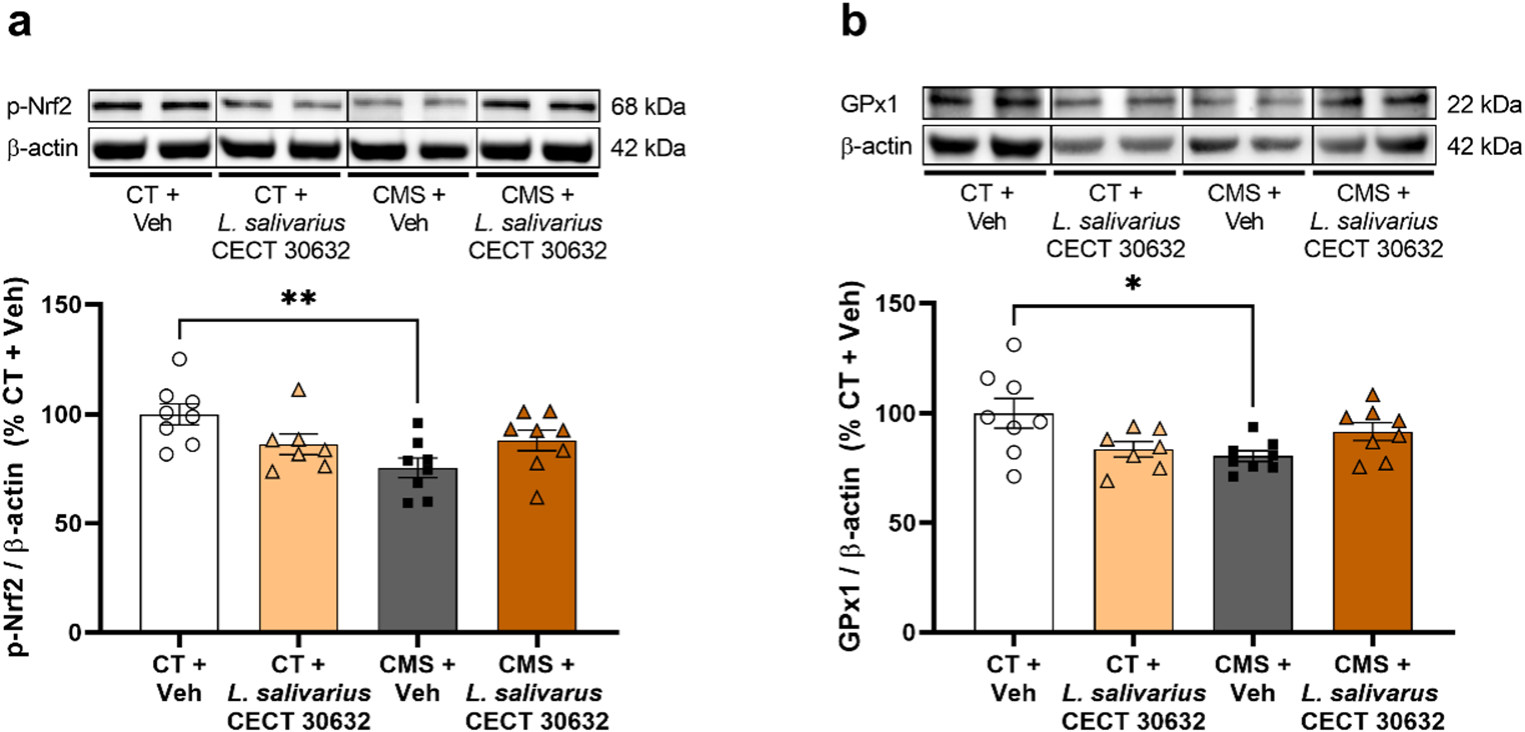
Antioxidant effects of *L. salivarius* CECT 30632 after chronic mild stress (CMS) in peripheral blood mononuclear cells (PBMCs). *L. salivarius* CECT 30632 prevented the CMS-induced reduction in the protein expression of phospho-nuclear factor (erythroid-derived 2)-like 2 (p-Nrf2) (**a**) and glutathione peroxidase 1 (GPx1) (**b**) in PBMCs, assessed by western blot. The densitometric data of the band of interest were normalized to that of beta-actin (β-actin). The data are presented as the means ± SEMs. *p < 0.05, **p < 0.01. One-way ANOVA test followed by Tukey’s post hoc. Blots were cropped (black lines) to improve the clarity and conciseness of the presentation

p-Nrf2 protein expression on PBMCs decreased in CMS + Veh compared to CT + Veh (*p* < 0.01), while this effect is blunted in the *L. salivarius* CECT 30632 groups (Fig. 3a). Similarly, the CMS + Veh group showed lower protein levels of GPx1 on PBMCs compared to CT + Veh (*p* < 0.05) and they were recovered in the CMS + *L. salivarius* CECT 30632 group (Fig. 3b).

### Modulation of the kynurenine pathway by *L. salivarius* CECT 30632 on plasma and FC after CMS

**Fig. 4:**
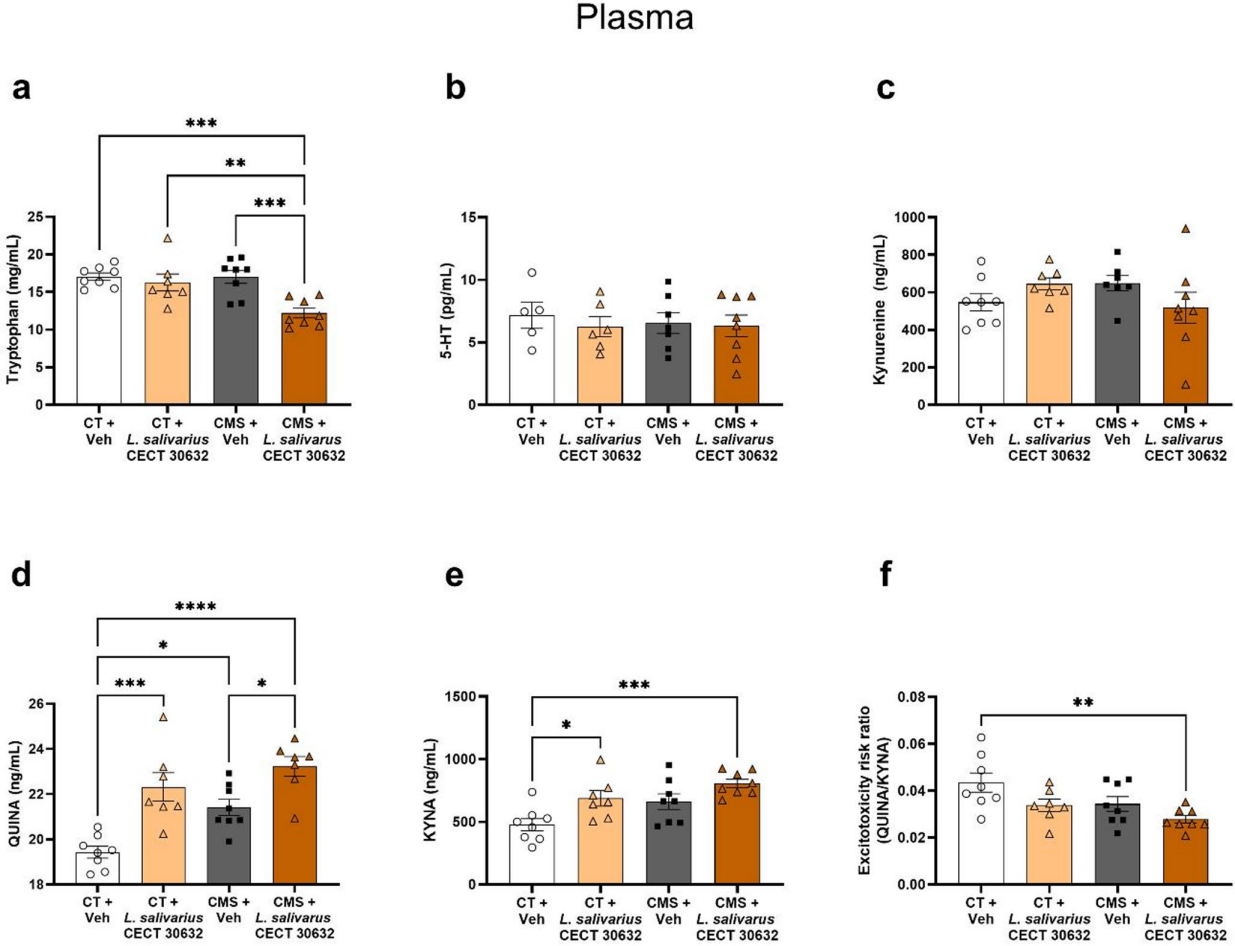
Modulation of the kynurenine pathway by *L. salivarius* CECT 30632 after chronic mild stress (CMS) in plasma. Enzyme-linked immunosorbent assay (ELISA) revealed lower tryptophan plasma levels in the CMS + *L.salivarius* CECT 30632 group (**a**), with no significant changes in serotonin (5-HT) (**b**), or kynurenine levels (**c**). Quinolinic acid (QUINA) plasma levels were increased in all experimental groups compared with CT + Veh group (**d**), whereas higher levels of kynurenic acid (KYNA) were observed only in *L.salivarius* CECT 30632-treated groups (**e**). The ratio between QUINA and KYNA (QUINA/KYNA), calculated as a theoretical excitotoxicity risk index, was reduced in the CMS + *L.salivarius* CECT 30632 group (**f**). The data are presented as the means ± SEMs. *p < 0.05, **p < 0.01, ***p < 0.001, ****p < 0.0001. One-way ANOVA test followed by Tukey’s post hoc

Plasma tryptophan levels were lower in the CMS + *L. salivarius* CECT 30632 group compared to CT + Veh (*p* < 0.001), CT + *L. salivarius* CECT 30632 (*p* < 0.01), and CMS + Veh (*p* < 0.001) (Fig. 4a). Plasma 5-HT and kynurenine levels did not change under our experimental conditions (Fig. 4b, c). There were higher levels of QUINA in the CT + *L. salivarius* CECT 30632 (*p* < 0.001), CMS + Veh (*p* < 0.05), and CMS + *L. salivarius* CECT 30632 (*p* < 0.0001) groups than in the CT + Veh group, and they were increased in CMS + *L. salivarius* CECT 30632 (*p* < 0.05) compared to CMS + Veh (Fig. 4d). Higher KYNA levels were found in the CT + *L. salivarius* CECT 30632 (*p* < 0.05) and CMS + *L. salivarius* CECT 30632 (*p* < 0.001) groups compared to the CT + Veh groups (Fig. 4e). The ratio between QUINA and KYNA, which can serve as a plausible index of the excitatory risk due to their antagonistic actions on the NMDA receptors [37], was significantly lower in the CMS + *L. salivarius* CECT 30632 (*p* < 0.01) group compared to CT + Veh (Fig. 4f).

**Fig. 5:**
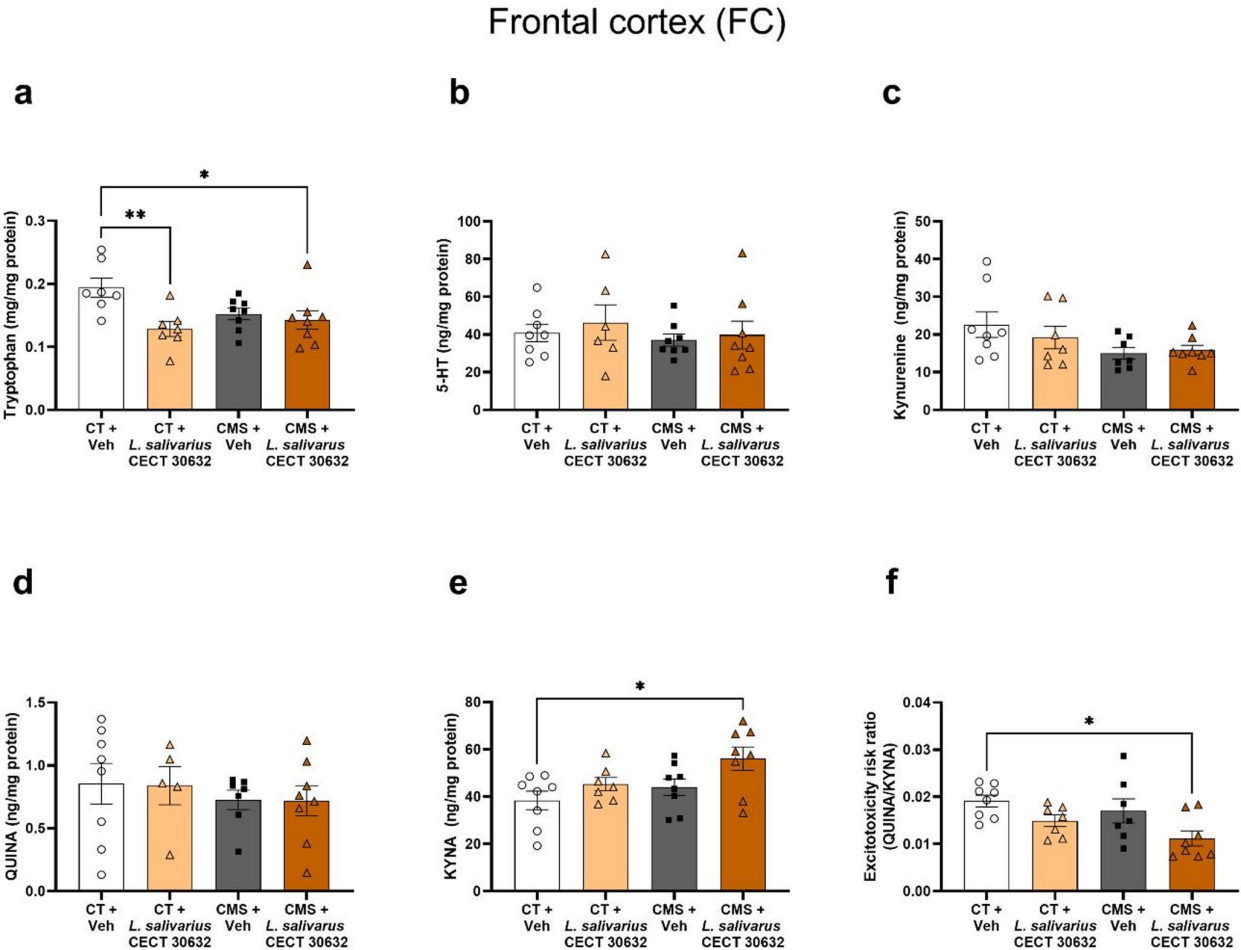
Modulation of the kynurenine pathway by *L. salivarius* CECT 30632 after chronic mild stress (CMS) in the frontal cortex (FC). Enzyme-linked immunosorbent assay (ELISA) revealed lower tryptophan FC levels of in *L.salivarius* CECT 30632-treated groups (**a**), with no significant changes in serotonin (5-HT) (**b**), or kynurenine levels (**c**). Quinolinic acid (QUINA) FC levels did not significantly change across all experimental groups (**d**), whereas higher levels of kynurenic acid (KYNA) were observed only in the CMS + *L.salivarius* CECT 30632 group (**e**). The ratio between QUINA and KYNA (QUINA/KYNA), calculated as a theoretical excitotoxicity risk index, was reduced in the CMS + *L.salivarius* CECT 30632 group (**f**). The data are presented as the means ± SEMs. *p < 0.05, **p < 0.01. One-way ANOVA test followed by Tukey’s post hoc (**a-c**,** e**). Kruskal–Wallis test with Dunn’s multiple comparisons (**d**,** f**)

In the FC, the tryptophan levels were lower in the CMS + *L. salivarius* CECT 30632 (*p* < 0.05) and in the CT + *L. salivarius* CECT 30632 (*p* < 0.01) groups compared to the CT + Veh (Fig. 5a). No changes for 5-HT, kynurenine, and QUINA were detected (Fig. 5b-d). The CMS + *L. salivarius* CECT 30632 group showed higher levels of KYNA (*p* < 0.05), and a lower excitotoxicity risk (*p* < 0.05) compared to CT + Veh (Fig. 5e, f).

## Discussion

Our results revealed the effects of *L. salivarius* CECT 30632 on rats exposed to CMS which might be relevant to the treatment of stress-related disorders such as anxiety or depression. *L. salivarius* CECT 30632 administration in a CMS procedure improves behavioral outcomes, including reduced anhedonia and enhanced self-care, promotes antioxidant activity in PBMCs, and reduces the risk of excitotoxicity through modulation of kynurenine metabolite balance.

### *Ligilactobacillus salivarius* CECT 30632 improves anhedonia and self-care behaviors while preserving safety profile

The reduced latency time in the ST observed both on control and CMS groups supplemented with *L. salivarius* CECT 30632 aligns with the antidepressant properties of this bacterial treatment. Notably, several available antidepressants with different mechanism of action such as desipramine [35], escitalopram [38], fluoxetine [39], paroxetine [40], and desvenlafaxine [41], have demonstrated similar effects on grooming behavior in rodent models. Conversely, *L. salivarius* CECT 30632 did not affect anxiety and locomotion parameters in the OF test. There remains some debate regarding the anxiolytic effects of the *Lactobacillus* genus, with a meta-analysis highlighting that these effects are strain-specific and inconclusive for *L. salivarius* [42]. Nonetheless, the absence of the impact in the OF reinforces the safety of *L. salivarius* CECT 30632 as a potential probiotic, which is particularly important given that certain microorganisms have been reported to exhibit anxiogenic [43] or hyperlocomotive [44] properties.

To further evaluate the safety and non-anxiogenic profile of *L. salivarius* CECT 30632 administration, we analyze corticosterone levels — the primary hormone involved in the stress response in rodents — within our experimental setting. No statistically significant differences were observed (see Supplementary file, Table S2). However, the combination of diverse stressors [45, 46] and the chronic nature of the CMS model [47] often results in variability in corticosterone levels, a well-recognized limitation of this protocol. Consequently, this model may not be optimal for assessing the potential effects of *L. salivarius* CECT 30632 on this stress hormone. Notably, hypercortisolism is not a consistent hallmark of depression [48, 49].

To investigate the impact of *L. salivarius* CECT 30632 on hypothalamic-pituitary-adrenal axis activation, we conducted a pilot experiment using acute restraint stress. Corticosterone levels in stressed animals pretreated with *L.salivarius* CECT 30632 were comparable to the mean levels observed in the control group (see Supplementary file, Table S3). These preliminary findings confirm the absence of adverse effects following *L. salivarius* CECT 30632 administration and suggest a potential modulation of stress-induced corticosterone elevations. Nonetheless, further research with larger sample sizes is necessary to validate this hypothesis.

Unexpectedly, no significant differences were observed between the CMS + Veh and CT + Veh groups in the behavioral tests. Increased latency or decreased total grooming duration in the ST are generally considered reliable indicators of anhedonia and low self-care following CMS [34, 50, 51]. Likewise, reduced time spent in the center of the OF has been reported in animals subjected to CMS [52, 53]. A plausible explanation for the absence of these expected differences in our experimental setting could be the cow milk used as a vehicle, intended to mimic potential probiotic administration in humans and enhance the translational relevance of the findings. In rats, cow milk forms curds in the stomach, leading to delayed digestion and gastric emptying, which may affect nutrient absorption and alter the microbial balance [54]​. Additionally, cow milk consumption has been shown to increase body weight in rodents, impacting metabolism [55]. These physiological changes could influence the MGB axis and subsequently affect behavior [56, 57].

Consistent with this, the inclusion of an additional control group receiving water instead of cow’s milk revealed that milk administration was associated with increased body weight gain and longer ST latency in control animals (Figure S1). In contrast, no group differences were observed in the OF test. These data suggest that cow’s milk may induce a shift in some baseline physiological and behavioral values, potentially attenuating the detection of CMS-induced alterations when comparisons are restricted to milk-treated groups. Importantly, despite this baseline shift, the typical CMS-associated reduction in body weight gain was preserved, and the effects attributed to *L. salivarius* CECT 30632 remained evident when evaluated relative to the vehicle While further experiments specifically designed to validate vehicle effects are required, the antianhedonic and self-care–promoting effects of *L. salivarius* CECT 30632 are notable, as they are observed under both control and chronic stress conditions, even in the presence of a potentially confounding vehicle.

### *Ligilactobacillus salivarius* CECT 30632 restores the cellular antioxidant system

Increased peripheral oxidative stress is a consistent finding in depression [58]. In PBMCs, the activated form of the cellular master antioxidant regulator p-Nrf2 and the antioxidant enzyme GPx1 were reduced in the CMS + Veh group. Similar results have been observed in the brain following CMS [59–61] and in patients with depression [62], indicating an impaired antioxidant system associated with the disorder. Moreover, a meta-analysis identified downregulation of Nrf2 in both preclinical and clinical studies of depression, further demonstrating that upregulation of these pathways is linked to antidepressant responses [63]. Notably, *L. salivarius* CECT 30632 administration mitigated the CMS-induced reduction in p-Nrf2 and GPx1, restoring their expression closer to control levels. This capacity to preserve a healthy antioxidant system could represent a beneficial mechanism for treating depressive disorders.

### *Ligilactobacillus salivarius* CECT 30632 modulates kynurenine metabolites in plasma and frontal cortex, potentially reducing excitotoxicity risk

At the peripheral and central levels, kynurenine pathways were analyzed due to its potential involvement in depression [64]. CMS exposure did not alter plasma levels of tryptophan, 5-HT, or kynurenine. Previous studies have reported inconsistent changes in these molecules following CMS, highlighting the significance of downstream effects on kynurenine pathways [65, 66]. Tryptophan levels were reduced in the CMS + *L. salivarius* CECT 30632 group, likely related to the increased QUINA and KYNA levels. The decrease in tryptophan did not affect circulating concentrations of 5-HT or kynurenine, maintaining 5-HT bioavailability. The absence of detectable changes in kynurenine levels in our samples may be explained by temporal dynamics, whereby tryptophan-derived kynurenine had already been further metabolized into QUINA and KYNA at the time of tissue collection, precluding the detection of transient increases in kynurenine itself. Beyond the kynurenine and serotonergic pathways, tryptophan can also be metabolized through alternative routes that were not directly assessed in the present study, including microbial conversion into indole-derived compounds. Several *Lactobacillus* species are known to catabolize dietary tryptophan into bioactive indoles derivatives capable of activating the aryl hydrocarbon receptor, thereby modulating host immune responses and gut–brain signaling [67, 68]. Thus, the observed reduction in tryptophan levels in *L. salivarius* CECT 30632-treated animals may reflect a redistribution of tryptophan toward alternative metabolic pathways. Although these hypotheses remain speculative, they provide plausible mechanistic frameworks that warrant further investigation.

Despite the increase in the neurotoxic kynurenine metabolite QUINA following CMS, there was no corresponding rise in excitotoxicity risk, as previously reported [69]. In patients with bipolar disorder experiencing a depressive episode, the QUINA/KYNA ratio is elevated in plasma and has been proposed as a biomarker for active depressive states, though this pattern is not seen in patients diagnosed with major depressive disorder [70]. Notably, *L. salivarius* CECT 30632 administration increased both QUINA and KYNA while reducing the excitotoxicity risk ratio, an effect associated with antidepressant treatments such as desipramine, escitalopram, and duloxetine [37].

In the FC, CMS did not affect the levels of the measured metabolites. We have previously reported alterations in QUINA and KYNA, suggesting a shift toward a harmful excitatory profile in this brain region following CMS [37]. As discussed for behavioral results, the use of cow milk as a vehicle might have interfered with the typical CMS effects on some parameters. Further research is clearly needed to validate this hypothesis. Nevertheless, a similar pattern was observed in the brain as in the periphery regarding the effects of *L. salivarius* CECT 30632 on kynurenine pathways. Under CMS conditions, *L. salivarius* CECT 30632 administration decreased the excitotoxicity risk ratio by selectively increasing KYNA in this case. Again, the reduced tryptophan levels appeared to be associated with the increase in KYNA rather than any adverse effects on 5-HT. A systematic review has warned about the lack of consistency in kynurenine metabolite measurements between brain and blood [71]. Our CMS + Veh data underscore the need for further investigation to clarify the role of kynurenine pathways in the CMS model, but they also demonstrate the potential of *L. salivarius* CECT 30632 to promote beneficial actions on these pathways at both central and peripheral levels.

Clinical studies reinforce the potential involvement of kynurenine pathways in depressive disorders, particularly through its relevance as a source of biomarkers. In patients with treatment-resistant depression undergoing electroconvulsive therapy, antianhedonic response may be predicted by circulating kynurenine metabolites levels [72]. Suicidal ideation has also been associated with a higher proportion of kynurenine versus tryptophan in these patients, a finding that correlates with inflammatory markers [73]. Notably, the mechanistic link between inflammatory stimuli and alterations in kynurenine metabolism has been demonstrated in humans, as LPS administration induces both QUINA and KYNA in humans [74]. The induction of these metabolites under an inflammatory atmosphere is consistent with our findings in the preclinical model.

The identification of QUINA as neurotoxic and KYNA neuroprotective in stress-related disorders has been supported in rodents by a recent meta-analysis [75]. However, this type of studies in humans have mainly identified only an association between depression and KYNA levels [64], while others suggest that this relationship, if present, is weak [76]. The potential role of KYNA in depression is reinforced by antidepressant and anti-inflammatory drugs promoting KYNA levels [77]. However, the causal connection between kynurenine metabolites and depressive symptomatology remains controversial [78], and clinical evidence for therapies directly targeting kynurenine pathway modulation in humans is limited. To date, only two clinical trials based on this strategy are registered in ClinicalTrials.gov. Both evaluate the administration of L-leucine, an essential amino acid that competitively inhibits the kynurenine transport into the brain and may therefore exert neuroprotective effects. One of these trials was terminated before completion and reported unconclusive results due to a very small sample size (*n* = 4) [79], whereas the other is currently recruiting, with an estimated completion date in 2029 [80].

## Conclusion

*Ligilactobacillus salivarius* CECT 30632 demonstrated beneficial effects in rats exposed to CMS, improving behavioral outcomes related to anhedonia and self-care, enhancing antioxidant activity, and modulating kynurenine pathways to reduce excitotoxicity risk. These findings suggest that *L. salivarius* CECT 30632 may have antidepressant properties without inducing anxiogenic or hyperlocomotive effects, reinforcing its safety profile as a probiotic treatment for stress-related disorders.

Our experimental design presents certain limitations that must be acknowledged. The exclusive use of male rats introduces a sex-related bias, which is particularly relevant in light of emerging evidence supporting sex differences in the MGB axis and kynurenine metabolism. Moreover, high intragroup variability in the behavioral tests might hinder some effects due to the limited sample size. Future studies including both sexes and increasing the sample size will be essential to strengthen the robustness and generalizability of our findings to depressive populations. Additionally, future research should explore the potential interactions between probiotics and different vehicles, such as cow milk, which may influence behavioral and physiological outcomes. Further investigation into the kynurenine pathway’s role in depression, both centrally and peripherally, will also be essential for validating the therapeutic effects of *L. salivarius* CECT 30632 and optimizing its application in clinical settings.

## Supplementary Information

Below is the link to the electronic supplementary material.

Supplementary Material 1

## Data Availability

The data that support the findings of this study are openly available in Docta Complutense at https://hdl.handle.net/20.500.14352/118406.
