# Supplementary material for: Behavioral, antioxidant, and kynurenine pathway modulation of a specific strain of Ligilactobacillus salivarius in a preclinical model of depression
Source: Eur J Nutr. 2026 Mar 9;65(3):86. doi: 10.1007/s00394-026-03941-9 (PMC12971813; doi:10.1007/s00394-026-03941-9)
Supplement: Supplementary file 1 — Supplementary Material 1 [file 394_2026_3941_MOESM1_ESM.docx]

**SUPPLEMENTARY FILE**

## Title: **Behavioral, antioxidant, and kynurenine pathway modulation of a specific strain of *Ligilactobacillus salivarius* in a preclinical model of depression**

**Author information:** David Martín-Hernández^a,b^*^#^, Javier R. Caso^a,b^*, César Díaz-García^a,b^, Pedro-Antonio Regidor^c^, José Miguel Rizo^d^, Marta Román^d^, Rocío Gutiérrez^d^, Juan C. Leza^a,b^

* These authors contributed equally to this work

**Correspondence (#)**

Dr. David Martín Hernández

Department of Pharmacology and Toxicology, School of Medicine, Complutense University,

Madrid. Pza. Ramón y Cajal s/n, 28040 Madrid, Spain.

Phone: +34 635564032

e-mail: [davidmhbiotec@gmail.com](mailto:davidmhbiotec@gmail.com)

**Western blot antibodies: technical details**

| Target | Catalog number | Manufacturer | Dilution |
| --- | --- | --- | --- |
| phospho-Nrf2 (Ser40) | 15882704 | Fisher Scientific | 1:1000 |
| GPx-1 | bs-3882R | Bioss | 1:1000 |
| Goat anti-rabbit IgG-HRP | sc-2357 | Santa Cruz Biotechnology | 1:2000 |

**Table S1:** Technical details of the antibodies used in western blot analyses.

**Corticosterone plasma levels following chronic mild stress (CMS) and *Ligilactobacillus salivarius* CECT 30632 administration**

Corticosterone plasma levels were measured using a commercially available ELISA kit (ENZO Life Sciences, Farmingdale, NY, USA) according to the manufacturer's instructions.

Chronic mild stress (CMS) and *L. salivarius* CECT 30632 administration did not modify corticosterone levels (one-way ANOVA, F(3,23)=1.430, p=0.0610) (**Table S2**).

| Experimental group | Mean corticosterone (ng/mL) | 95% CI |
| --- | --- | --- |
| CT + Veh | 63.43 | [49.39, 77.47] |
| CT + *L. salivarius* CECT 30632 | 88.39 | [50.35, 126.4] |
| CMS +Veh | 54.71 | [25.45, 83.97] |
| CMS + *L. salivarius* CECT 30632 | 43.02 | [31.54, 54.49] |

**Table S2:** Corticosterone plasma levels following chronic mild stress (CMS) and *L. salivarius* CECT 30632 administration.

**Pilot experiment to assess the effects of *Ligilactobacillus salivarius* CECT 30632 on corticosterone levels following acute restraint stress**

This study adhered to the modified ARRIVE guidelines 2.0 for preclinical *in vivo* research and to Spanish and European Union regulations (RD 53/2013 and EU Directive 2010/63/EU for animal experiments). The experimental protocol was approved by the proper administrative authorities (PROEX 087/18) and conducted at the UCM Animal Facility of the Complutense University of Madrid.

Male Wistar Hannover rats (HsdRccHan: Wist, Envigo, Spain), weighing approximately 300 g, were housed under controlled conditions: a constant temperature of 24 ± 2°C, relative humidity of 70 ± 5%, and a 12-hour light‒dark cycle (lights on at 8:00 AM). Before the experiments, the animals were acclimated to these conditions and handled daily for seven days, with unrestricted ad libitum access to fresh filtered tap water and standard pellet chow (A04 SAFE, Scientific Animal Food and Engineering, Augy, France) throughout the experimental procedures.

Rats were randomly assigned to three experimental groups: CT (n=6), S6h + Veh (n=4), and S6h + *L. salivarius* CECT 30632 (n=5). Vehicle/placebo (skimmed milk, 1mL) or *Ligilactobacillus salivarius (L. salivarius)* CECT 30632 (1x10^10^ colony forming units (CFU) dissolved in 1mL of skimmed milk) was administered daily by gavage for 2 weeks prior to the stress protocol. Acute restraint stress model consisted of six hours of immobilization (S6h) using a plastic rodent restrainer designed for a close fit. While the stressed rats were restrained, food and water access were removed from the control group. The stress protocol began at 9:00 AM, and blood samples were collected immediately after the restraint session (between 2:00 and 3:00 AM to avoid the influence of circadian changes in the stress response) following terminal anesthesia with sodium pentobarbital (220 mg/kg i.p. Vetoquinol®, Madrid, Spain).

Blood was obtained via cardiac puncture, anticoagulated with 1% w/v ethylenediaminetetraacetic acid (EDTA) (1 volume EDTA per 50 volumes blood), and centrifuged at 1500 rpm for 15 minutes to obtain plasma. Corticosterone levels were measured using a commercially available ELISA kit (ENZO Life Sciences, Farmingdale, NY, USA) according to the manufacturer's instructions.

In our pilot experiment, corticosterone levels did not show statistically significant differences among the three experimental groups (one-way ANOVA, F(2,12)=1.430, p=0.2773). However, corticosterone levels in stressed animals pretreated with *L.salivarius* CECT 30632 were comparable to the mean levels observed in the control group (**Table S3**).

| Experimental group | Mean corticosterone (ng/mL) | 95% CI |
| --- | --- | --- |
| CT | 249.1 | [157.9, 340.3] |
| S6h +Veh | 344.8 | [123.8, 565.7] |
| S6h + *L. salivarius* CECT 30632 | 269.7 | [251.6, 287.8] |

**Table S3:** Corticosterone plasma levels following acute restraint stress (S6h) and *L. salivarius* CECT 30632 administration.

**Assessment of cow milk interference with the effects of *L. salivarius CECT 30632* on body weight gain, the splash test (ST), and open field (OF)**


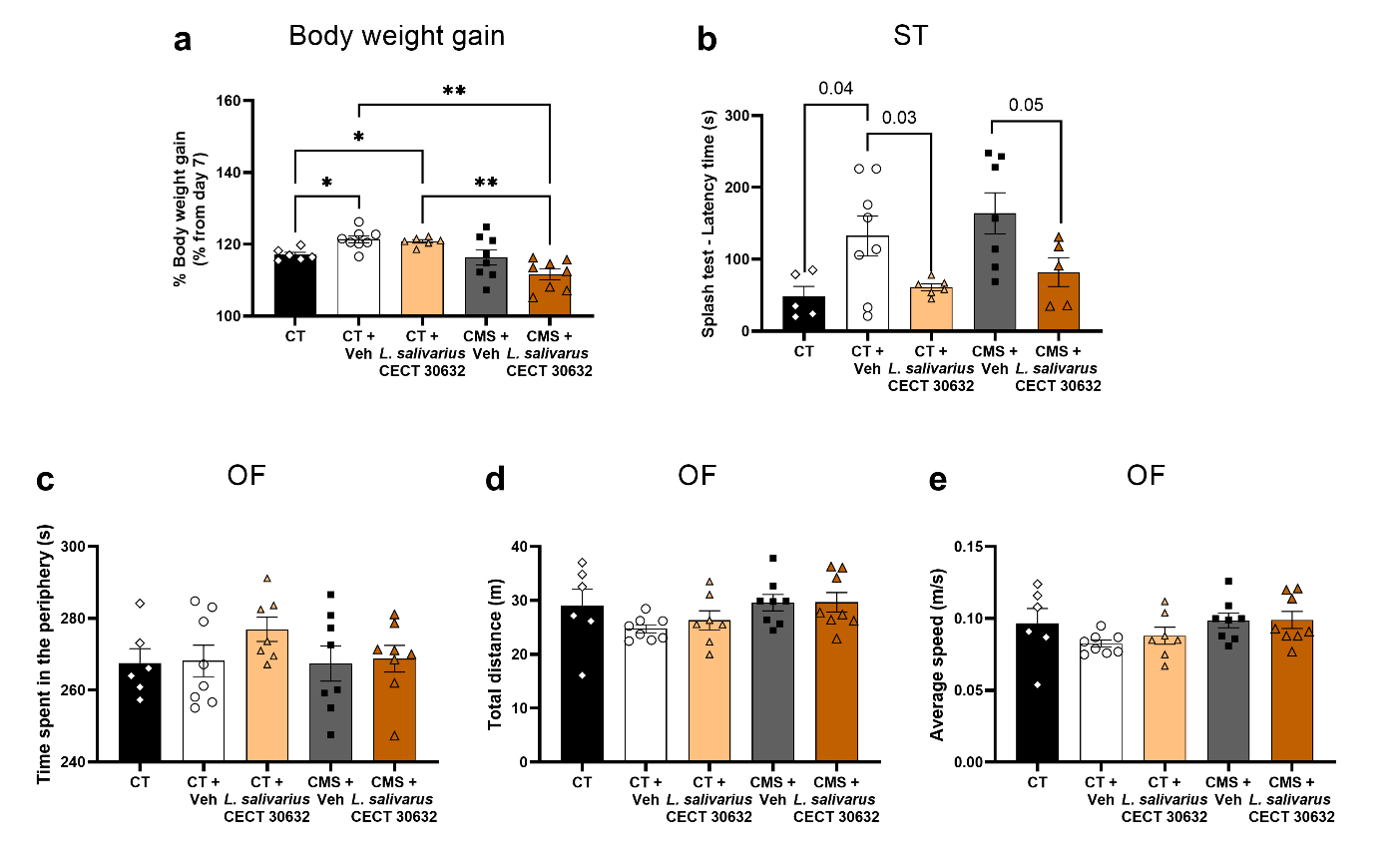
**Figure S1. Analysis of the possible interference of cow milk with the effects of *L. salivarius* CECT 30632 after chronic mild stress (CMS), assessed by body weight gain, the splash test (ST), and open field (OF).** Under control conditions, both vehicle and *L. salivarius* CECT 30632 increased the percentage of body weight gain from day 7 (CMS starting point) compared with the control group, whereas the CMS + *L. salivarius* CECT 30632 group showed a lower percentage of body weight gain compared with CT + Veh and CT + *L. salivarius* CECT 30632 (**a**). Under control conditions, vehicle administration increased ST latency time compared with control, whereas *L. salivarius* CECT 30632 administration decreased latency time under both control and CMS conditions (**b**). No effects on the OF parameters: time spent in the periphery (**c**), total distance (**d**), and average speed (**e**). The data are presented as the means ± SEMs. **p*<0.05, ***p*<0.01. *p*-value of t-test paired comparisons are indicated with numbers. Brown–Forsythe ANOVA test followed by Dunnett’s T3 post hoc test (**a,b**). One-way ANOVA test followed by Tukey’s post hoc (**c-e**).
